# Bursts and horizontal evolution of DNA transposons in the speciation of pseudotetraploid salmonids

**DOI:** 10.1186/1471-2164-8-422

**Published:** 2007-11-16

**Authors:** Johan G de Boer, Ryosuke Yazawa, William S Davidson, Ben F Koop

**Affiliations:** 1 Centre for Biomedical Research, University of Victoria, Victoria, BC V8W 2Y2 Canada; 2 Department of Molecular Biology & Biochemistry, Simon Fraser University, Burnaby, BC Canada

## Abstract

**Background:**

Several genome duplications have occurred in the evolutionary history of teleost fish. In returning to a stable diploid state, the polyploid genome reorganized, and large portions are lost, while the fish lines evolved to numerous species. Large scale transposon movement has been postulated to play an important role in the genome reorganization process. We analyzed the DNA sequence of several large loci in *Salmo salar *and other species for the presence of DNA transposon families.

**Results:**

We have identified bursts of activity of 14 families of DNA transposons (12 Tc1-like and 2 piggyBac-like families, including 11 novel ones) in genome sequences of *Salmo salar*. Several of these families have similar sequences in a number of closely and distantly related fish, lamprey, and frog species as well as in the parasite *Schistosoma japonicum*. Analysis of sequence similarities between copies within the families of these bursts demonstrates several waves of transposition activities coinciding with salmonid species divergence. Tc1-like families show a master gene-like copying process, illustrated by extensive but short burst of copying activity, while the piggyBac-like families show a more random copying pattern. Recent families may include copies with an open reading frame for an active transposase enzyme.

**Conclusion:**

We have identified defined bursts of transposon activity that make use of master-slave and random mechanisms. The bursts occur well after hypothesized polyploidy events and coincide with speciation events. Parasite-mediated lateral transfer of transposons are implicated.

## Background

Two genome duplications are thought to have occurred in the evolutionary history of ancient vertebrates, a third in ancestral teleosts, with a fourth occurring more recently in the genome of salmonid fishes (*Salmonidae*), 25 to 100 million years ago (Mya) [1,2]. After duplication and subsequent mutation of the duplicated chromosomes, the polyploid genome began to reduce to a more stable diploid form, losing approximately 50 percent of its DNA [3]. In this model, rediploidization preceeded and is concurrent with speciation events of the *Salmonidae *line to *Salmoninae *(salmon and trout), *Coregoninae *(whitefish), and *Thymallinae *(grayling). Subsequently, *Salmoninae *radiated into at least seven genera, including *Salmo *(Atlantic salmon and trout), *Oncorhynchus *(Pacific salmon and trout), and *Salvelinus *(char and brook trout), comprising of approximately 30 species [4-6]. The mechanism by which this restabilization occurs is not well understood, but large scale movement of repetitive sequences, in particular transposable elements, are postulated to play a major role in reshaping genomes and the genome's evolution. These sequences, which make up a large fraction of the genomes of plants and animals, can move about in the genome by making copies of themselves or by excision and reintegration. Significant increases in transposon activity have indeed been noted in plants after allopolyploidization [7], during chromosome rearrangements in Drosophila [8], and after species hybridization [9].

One type of these transposable elements, the DNA transposon, is completely excised from the genome and reintegrates in a different place. DNA transposons contain a single open reading frame that codes for a transposase enzyme. This enzyme facilitates the excision and reinsertion of the transposon element. In addition to moving through the genome of the host cell, transposons also appear to be able to move between species, in a process called horizontal or lateral transfer [10,11]. When an organism acquires a new active transposon, a burst of transposition activity may ensue until all copies are mutationally inactivated. Movement of large numbers of transposons through the genome can have profound consequences [12] as they may jump into genes or into controlling sequences and effectively become a mutagen, both *in vivo *[13] and *in vitro *[14].

Recombination between mobile elements may result in deletions, translocations, or the formation of dicentric chromosomes [15]. Such mobile elements, therefore, have helped shape genomes throughout evolutionary history. The mining of genomes for these elements and their subsequent phylogenetic analysis of members of same or different classes may shed light on these events. In this study we have recovered and identified the sequence features of approximately 250 members of 14 DNA transposon families in the salmonid *Salmo salar*. A number of these are also found in *Oncorhynchus mykiss *and in several other closely and distantly related fish species, in frogs, as well as in the parasite *Schistosoma japonicum*.

## Results

### DNA transposon families

**Figure 1:**
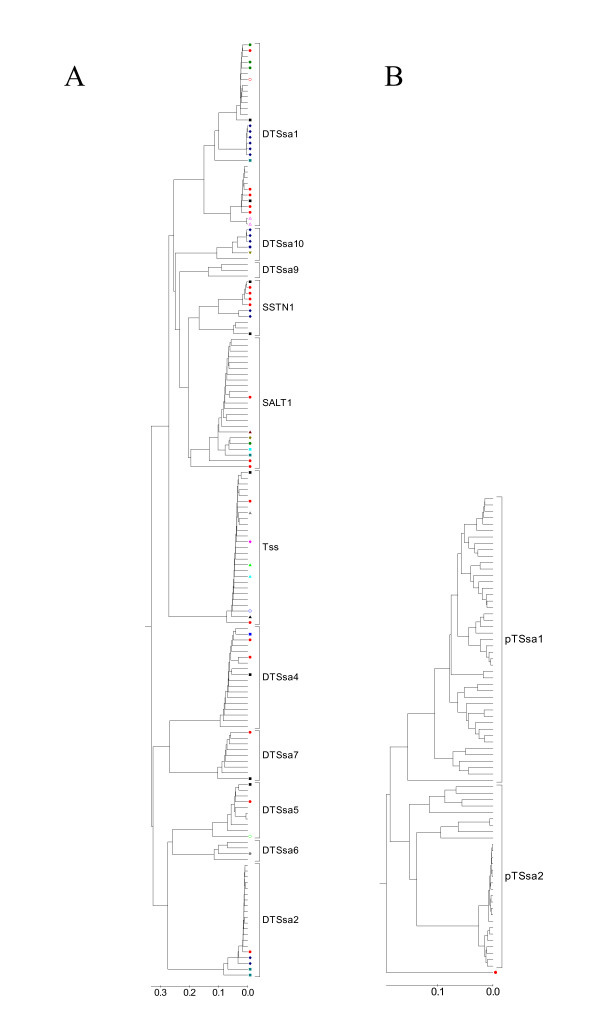
**Phylogenetic tree of DNA transposon sequences in *Salmo salar *and various other species**. A) Tc1-like DNA transposons; B) piggyBac-like DNA transposons. Sequence alignments were performed with ClustalW and phylogenetic trees generated with MEGA3.1 using the Unweighted Pair Group Method with Arithmetic Mean (UPGMA), pairwise deletion, and a p-distance model. Sequences used in the tree are at least 1000 bp, but EST sequences from *Schistosoma japonicum *are between 500 and 700 bp long. DTSsa3 is not included in the tree as only fragments of a few hundred base pairs were recovered that conform to the amino acid motifs required for a Tc1-like transposon. DTSsa5 has two copies that are closely related due to a recent duplication event. Shown below are the color markers for the species. No marker in the tree indicates *Salmo salar*. All others are: *Acanthophthalmus kuhli*; *Astatotilapia burtoni*; *Carassius auratus*; *Cyprinus carpio*; *Danio rerio*; *Deltistes luxatus*; *Esox lucius*; *Gasterosteus aculeatus*; *Ictalurus punctatus*; *Oncorhynchus kisutch*; *Oncorhynchus mykiss;  Oncorhynchus tsawytscha*; *Oryzias latipes*; *Petromyzon marinus*; *Polypterus bichir*; *Rana pipiens*; *Salvelinus fontinalis*; *Schistosoma japonicum*; *Tanichthys albonubus*; *Xenopus tropicalis*; *Xenopus laevis*.

To explore the role of transposons in speciation events in fish, nearly 3 Mbp of BAC sequence from *Salmo salar *as well as from *Oncorhynchus mykiss *was analyzed for the presence of DNA transposon-like sequences. In addition, a number of published clones from *Xenopus *and several other fish species were included in the analysis. We recovered over 250 fragments of transposon members from 14 different DNA transposon families (named DTSsa1 to pTSsa2) in the genome of *S. salar*, including a few possibly complete copies. Sequences are found in Additional Files 1 and 2. A total of 237 sequences were aligned in two separate groups, Tc1-like sequences (162 sequences), and piggyBac-like sequences (75 sequences). The sequences used are at least 1000 base pairs long, except for EST fragments from *S. japonicum*, which are 500 to 700 base pairs long. Phylogenetic trees were constructed separately for each of the two groups (fig 1). The sequence alignments are found in Additional Files 3 and 4. The DNA sequences of the families within each group are at least 20% different. In many instances, the recovered copies of transposons have internal deletions and insertions and frequently only fragments of an element were found.

Twelve families (figure 1A) belong to the Tc1/*mariner *class of transposons. This class is modeled on the Tc1 transposon in *C. elegans*, and members in this class are approximately 1,500 base pairs long, contain a single open reading frame encoding a transposase enzyme and end in two Inverted Terminal Repeats (ITR). The target specificity of Tc1 class elements dictates insertion at a genomic 5'-TA-3' dinucleotide sequence. The Tc1-like transposons we identified can be grouped into two subclasses (table 1). One subclass consists of sequences with long ITRs (~200 bp) and CAGT at the end of their inverted repeats (DTSsa family 1, 4, 9, 10 and SSTN1, Tss, and SALT1) while the other subclass has short ITRs (~20 bp) and variations of the CAGT sequence at their ends (DTSsa family 2, 5, 6, and 7). This dichotomy has been observed before [16,17]. Transposons SSTN1 [Genbank:AJ249090] [11], Tss [Genbank:L12207] [18], and SALT1 [Genbank:L22865] [19] have been described previously. There is insufficient sequence information for transposon DTSsa3 and DTSsa6 to determine the structure of the inverted terminal repeat sequences, although it is short for DTSsa6. However, there is partial sequence data for both that provides amino acid sequence motifs and confirms their nature as a DNA transposon. The DTSsa5 family has two copies that are highly similar (figure 1A) (< 0.1 percent difference) as a 15 kbp region in the TCR alpha locus in salmon, that contains one of the DTSsa5 copies, appears to have been recently duplicated.

**Table 1:** Features of the 14 DNA transposon families found in *Salmo salar*.

**Name**	**Insertion site**	**ITR end (4 bp)**	**ITR length (bp)**	**~Length (bp)**^**§**^	**Group**
DTSsa01	5'-TA-3'	CAGT	200	1600	Tc1
DTSsa02	TA	CACT	~40	1600	Tc1
DTSsa03	TA	ND	ND	ND	Tc1
DTSsa04	TA	CAGT	244	1530	Tc1
DTSsa05	TA	AACT	~18	1440	Tc1
DTSsa06	ND	ND	ND	ND	Tc1
DTSsa07	TA	CACT	~20	ND	Tc1
SSTN1	TA	CAGT	206	1570	Tc1
DTSsa09	TA	CAGT	212	ND	Tc1
DTSsa10	TA	CAGT	199	ND	Tc1
Tss	TA	CAGT	243	ND	Tc1
SALT1	TA	CAGT	204	ND	Tc1
pTSsa1	TTAA	CCTG	~22	1440	piggyBac
pTSsa2	TTAA	CCTG	~22	1440	piggyBac

§: Approximate length of sequences

ND: Not determined

**Figure 2:**
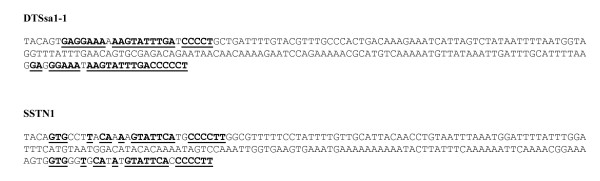
**Terminal repeat structure**. Inverted Terminal Repeat of examples of recovered members of the DTSsa1 (upper sequence) and SSTN1 (lower sequence) families. The TA insertion site is shown at the start of the sequences, followed by the canonical CAGT. The two direct repeated sequences at the two ends of an ITR are in bold and underlined.

The conservation of the left and right ITR of a transposon is not always easily identified. For example, while DTSSa1 has very conserved left and right ITRs, SSTN1 and SALT1 have much less conserved ITRs. However, the long ITRs have a direct repeated sequence located at their outer and inner ends [16], representing binding sites for the transposase enzyme. This makes it possible to identify the entire ITR through these direct repeats. For example, SSTN1 appears to have an 80 base pair ITR (judged by a dotter plot) and was reported as such [11], but this can be extended to 206 base pair when judged by the presence of the direct repeats (figure 2).

We identified two non-Tc1-like transposon families (pTSsa1 and pTSsa2; Table 1 and Fig 1B). Sequence characteristics suggest that they consist of piggyBac-like transposons, based on their genomic insertion specificity (5'-TTAA-3') and the very ends of the approximately 18 base pair ITR sequence (5'-CCT-3'; consistent with the 5'-CCY-3' of reported sequences) [20,21]. In addition, the left and right inverted terminal repeats of pTSsa1 and 14 have a consistent several nucleotide difference, which agrees with previous findings for piggyBac ITR sequences [22]. These two families contain a 380 base pair sequence which is known as the NheI repeat [GENBANK:L25409] [23]. Even though many very similar copies are present in salmon, the sequence itself does not appear to contain any significant open reading frames or recognizable amino acid motifs. A single copy, similar to these sequences, was also found in *Oncorhynchus mykiss *(in the IgH.A locus [GENBANK:AY872256]) (figure 1B). Interestingly, whereas the pTSsa1 and 14 families are only approximately 80 percent similar, the first 200 base pairs of these sequences share a higher degree of similarity (approximately 94 percent), possibly indicating a functionally conserved role for this region. The pTSsa1 and 14 sequences have been very active as seen in the high degree of similarity between many members. In particular, pTSsa2 has many nearly identical copies (< 0.1 percent different). In several instances copies have been found inside one another. In one example, a copy inserted itself into a TTAA sequence of a sequence containing the *S. salar *MHC class 1b locus, a second copy inserted itself at a TTAA sequence inside the first copy, and a third copy inserted itself into the second copy. Interestingly, the third copy belongs in a phylogenetic branch comprised of multiple nearly identical copies (pTSsa2), consistent with the most recent insertion event. In several other instances, a copy has been found inside another copy (*e.g*. in the salmon MHC class 1b locus), inserted into a DTSsa11 transposon in the salmon TCRα locus, and two copies were found perfectly adjacent in the salmon TCRα locus, with a shared 5'-TTAA-3' sequence between them.

### Transposon sequences are transcribed

DNA transposons have a single open reading frame, coding for a transposase enzyme. Active transposons would result in messenger transcripts of the gene. Krasnov et al. [24] suggested from expression microarray data that transposase genes were probably transcribed from their own promoters and that expression increased with environmental stress. We searched our salmon EST database [25,26] for the presence of transcripts that contain DTSsa transposon sequences. Perhaps surprisingly, representatives all of the DTSsa transposon families are represented in the EST transcripts. One representative sequence of each transposon was used in the search, resulting in many EST hits. We found a similar variation in the recovered EST sequences for a DTSsa family as we find in the genomic sequence within each DTSsa family, indicating that many different members of each family are present among the transcripts (data not shown). However, many of the transposon families (*e.g*. DTSsa1, DTSsa2, Tss, SALT1, and pTSsa1 and pTSsa2) were found as complete copies (*i.e*. including their ITRs) internal to transcripts present in the salmon EST database. While transcription from internal transposon promoters cannot be excluded, these transcripts, therefore, are initiated from a sequence adjacent to the transposon, and not from an internal transposon promoter. This indicates that the transposons have inserted themselves into actively transcribed genes.

### Similar DNA transposons in other species

Using the DNA transposons found in salmon as query sequences for Genbank BLAST searches, we have recovered similar sequences from numerous different fish and frog species (table 2). Interestingly, many of the sequences recovered from different species can be aligned with the salmon Tc1-like sequences and placed in the phylogenetic tree in such a way as being indistinguishable from those recovered from *S. salar *(figure 1). This includes sequences from such diverse species as pike, *Polypterus*, and the frogs *Rana pipens*, *Xenopus laevis *and *Xenopus tropicalis*.

**Table 2:** Recovered transposon families found in sequences from other species.

**Organism**	**Family**	**Genbank accession numbers**				
*Acanthophthalmus kuhli*	Tss	L48686				
*Astatotilapia burtoni*	DT1	DQ386647	L41173			
*Carassius auratus*	Tss	AY351357				
*Chasmistes brevirostris*	Tss	AY351357				
*Cyprinus carpio*	Tss	L48683				
*Danio rerio*	DT1	CR753816				
*Danio rerio*	DT2	CR790363	CR376861			
*Danio rerio*	DT10	AL954144				
*Danio rerio*	SALT1	CR450768				
*Deltistes luxatus*	DT5	AF314683				
*Esox lucius*	Tss	L41172				
*Gasterosteus aculeatus*	SALT1	AC182753				
*Ictalurus punctatus*	DT1	DQ400445				
*Oncorhynchus kisutch*	Tss	DQ668034				
*Oncorhynchus mykiss*	DT1	AB162342				
*Oncorhynchus mykiss*	DT3	DQ246664				
*Oncorhynchus mykiss*	SSTN1	AB162343				
*Oncorhynchus mykiss*	DT13/14	AY872256				
*Oncorhynchus tshawytscha*	SALT1	AY100012				
*Oryzias latipes*	DT6	BA000027				
*Petromyzon marinus*	DT1	AF464190				
*Polypterus bichir*	DT10	AC132195				
*Rana pipens*	DT10	AY261371				
*Salvelinus confluentus*	DT7	AY788871				
*Salvelinus fontinalis*	DT4	AY308064				
*Salvelinus namaycush*	Tss	AF017232				
*Tanichthys albonubus*	Tss	L48685				
*Xenopus laevis*	DT10	U43662				
*Xenopus tropicalis*	DT1	AC145794	AC148453	AC148466	AC149518	AC149594
		AC149875	AC149881	AC155950	AC155952	
*Xenopus tropicalis*	DT2	AC147826	AC166141			
*Xenopus tropicalis*	DT10	AC147174				

Genbank accession numbers indicate the sequence(s) in which the element was found.

Most of the transposon families, with the exception of DTSsa9 and 10, and pTSsa1 and 2, were also identified in Genbank EST collections of *Schistosoma japonicum *(listed in Methods). These sequences, found in *S. japonicum *by Liu *et al*. [27], are very similar to those found in various fish species, and, when compared to similar sequence fragments, are placed close with members of different species. While no sequence similar to DTSsa10 has been found in *S. japonicum*, a 490 base pair common sequence fragment of DTSsa10 from salmon is also found in the frogs *Rana pipens*, *Xenopus laevis *and *Xenopus tropicalis *with at least 88 percent similarity.

## Discussion

**Figure 3:**
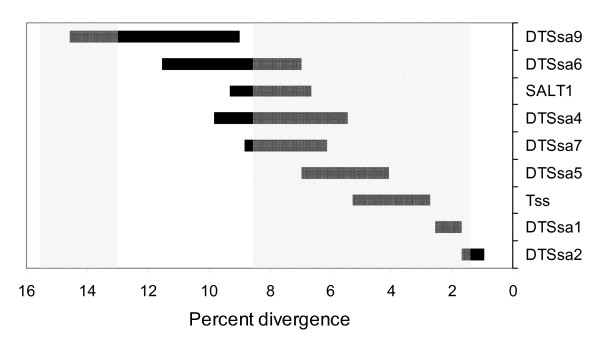
**Bursts of DNA transposon activity**. Horizontal bars represent the duration of bursts of Tc1-like DNA transposon in *Salmo salar*, based on earliest and latest branching. The shaded areas indicate the approximate timing of the genome duplication event (left) and further speciation events [4] (right).

Transposable elements have been implicated in genomic reorganizations. Our study investigates the possible connection between DNA transposition and the speciation process. To this end we have identified numerous members of many novel DNA transposons families in the salmonid *Salmo salar *as well as several in a number of related species. Of fourteen identified transposon families, eleven are novel. The remaining three are the previously described DNA transposons SALT1, Tss, and SSTN1. An analysis of the sequences revealed some interesting features and relationships. Phylogenetic grouping after alignment of the DTSsa and pTSsa transposon sequences based on complete or nearly complete DNA sequence [28] is shown in figures 1A and 1B. We have named the families based on branching at around 80–85 percent similarity. This approximately coincides with the proposed timing of the last genome duplication event [1,29,30]. The 14 transposon families include twelve Tc1-like families and two piggyBac-like families; approximately 250 copies and fragments of sequences from *Salmo salar *and *Oncorhynchus mykiss *and several other species. Members of the Tc1-like families have become active at different times in the past and then display a short period of intense copying activity (figure 1 and 3). The copying pattern seen for piggyBac-like transposons, however, exhibits a different, more random mechanism.

### Mode of transposition

The number of transposons in the genome increases in a manner depending on their copying mechanism. A retrotransposon is transcribed as RNA and subsequently reverse transcribed and integrated, creating a copy of itself. DNA transposons, on the other hand, excise themselves using their encoded transposase, and the element then reintegrates elsewhere in the genome. This results in movement of the transposon, rather than producing a copy. Mechanisms for increased copy number have been suggested, for example, the copy number can increase during S-phase if reinsertion occurs ahead of a replication fork.. Two models have been suggested to account for the appearance of a phylogenetic tree of copies of transposon sequences [31,32], a master gene model, in which one or several active sequences spawn inactive copies of themselves, and a random template model where many copies produce offspring which may subsequently become active themselves. Our data provide examples of both of these outcomes. The expansion patterns of the Tc1-like transposon families appear to follow a typical master-like copying mechanism. Most of the Tc1-like families in salmon "explode" in bursts of copying activity at different points in the past, but later seem to become quiescent (Fig. 1). Each burst of activity results in a sequential replacement pattern. On the other hand, the two piggyBac-like transposon families appear to follow a more random evolution pattern. This difference in propagation pattern points to a difference in transposition mechanism. Our finding of several families where the entire transposon sequence, including their ITRs, is present inside expressed EST sequences suggests a way of replication through retro-transcription, resulting in a "master gene" appearance of the phylogenetic tree of Tc1-like families. However, as this mechanism would not require an active DNA transposon, the cessation of the burst would depend on the transcription of the "host gene" becoming inactivated, something we do not observe. As well, the pTSsa1 and pTSsa2 families are also found as complete copies internal to EST transcripts, and these families do not display a typical "master gene" appearance. Whereas the Tc1-like families have coding potential for a transposase enzyme, there is no recognizable coding for any relevant amino acid motif in pTSsa1 and 2. As the first 200 base pairs of members of pTSsa1 and pTSsa2 are highly similar, this may suggest a functional role for these sequences. These elements are especially concentrated in the *S. salar *T-cell receptor alpha variable gene region, where small clusters of them are interspersed with small clusters of variable genes. Of interest is the very recent expansion of some of these elements of the pTSsa2 family (divergence < 0.1 %).

### Active transposons?

DNA transposons gather mutations over time, resulting in vertical inactivation [33]. Eventually all that remains are relics of the original sequence; the majority of DNA transposons in genomes are no more than fragments and altered sequences. Therefore, most instances are remnants, and only those that have recently been active can be found as intact or near intact copies. Transposon sequences that have displayed recent activity will have copies that are more similar, such as DTSsa1 and DTSsa2. A few copies of these transposon families have an open reading frame. One copy of DTSsa1 in salmon and two copies of DTXtr1 in *X. tropicalis *share 82% amino acid similarity over an open reading frame of 340 amino acids. One member of DTSsa2 has an open reading frame of 319 amino acids, while a copy of DTOmy2 has a single frameshift interrupting the reading frame. These reading frames share all the characteristics of Tc1-like transposase amino acid sequences which are typically between 320 to 343 residues long and contain the typical DD(34)E transposase motif [18]. Other transposon families that have gathered more mutations display much more sequence variation and except for stretches with recognizable amino acid motifs, no corresponding open reading frames have been found. It is therefore possible that both the DTSsa1 and DTSsa2 families currently contain active transposons. Sleeping Beauty has been reconstructed from a Tc1-like sequence, active > 10 Mya [34]. Since open reading frames were identified in DTSsa1 and DTSsa2, it suggests that it is possible to construct useful vectors from these more recent transposons.

### Horizontal invasion

Transposons can spread rapidly through a population. Elements of DNA transposon P spread through the entire *Drosophila melanogaster *population on all continents since the 1950's [35]. The finding that a nearly identical P element is present in *D. willistoni *strongly suggested a recent horizontal transfer between the two species [10]. *Mariner *elements have also been seen to transfer between *Diptera *and *Neuroptera *[36]. Leaver [11] reported the occurrence of SSTN1 in salmon, and similar sequences in flatfish and *Rana temporaria *and also suggested horizontal transmission between species. Radice et al [18], on the other hand, isolated transposon sequences from three teleost species and found no indication of similarities. Interestingly, we have identified highly similar sequences from numerous, rather diverse species. Sequences very similar to the DNA transposons in *Salmo salar *are found in various other fish species, including other teleosts as well as the sea lamprey *Petromyzon marinus *and the frogs *Xenopus *and *Rana*.

In particular, copies of Tss greater than 90% similar to those seen in salmonid members were found in eight different species, including the very distant northern pike *Esox lucius *which branched away before a genome duplication that led to the teleost line. The DTSsa10 sequence of *S. salar *is also found in very diverse species, including in the primitive bony fish *Polypterus bichir*, which branches off before the genome duplication of 400 Mya, and in the frogs *Rana pipens*, *Xenopus laevis *and *Xenopus tropicalis*, with more than approximately 88 percent similarity over a 490 base pair sequence. Confirming initial reports of fish transposons in the trematode worm parasite *Schistosoma japonicum *by Melamed *et al*. [37] and Matveev *et al*. [5], we have also found most transposon families in EST data of this parasite. We identified sequences of all the transposon families except the Tc1-like DTSsa9 and 10, and the piggyBac-like pTSsa1 and pTSsa2 in EST data of *S. japonicum*. We have included the eight longest sequences that were found in the parasite data, in six families, in the data for alignment and the phylogenetic tree (Fig. 1). The finding that most of the salmon transposon sequences are found in EST data of the parasite strongly suggests that this organism has provided a general shuttle service for lateral transposition between different aquatic species. Currently the related *Diplostomum *species, not *Schistosoma*, have fish as part of their life cycle. However, host switching is a feature of schistosome evolution [38] and a host-parasite relationship over evolutionary times cannot be ruled out. Our series of rapid bursts of expansion (fig 1 and 3) is in agreement with numerous horizontal invasions at different times in the past.

### Relation between transposition and speciation

A burst of speciation in the salmonid line followed a duplication of the teleost genome which happened approximately 25 to 100 Mya [1,2]. This duplication event is thought to have occurred at 86% similarity [29,39]. Our transposon naming system originates around this point. The *Salmonidae *line then branched into *Salmoninae*, *Coregoninae*, (and *Thymallinae*) around the 92% similarity point [40]. The subsequent divergence of the *Salmoninae *lineage into *Salmo*, *Oncorhynchus*, and *Salvelinus *occurred over a short time [41], and is estimated to have occurred between 14 to 23 million years ago [4,42]. This separation took place at 94–95% similarity [39]. Subsequent speciation continues to the present time. Bernatchez placed the most recent speciation events, including those to *S. salar *and *S. trutta*, between 0.5 and 2.0 million years ago [43], based on fossil data and mitochondrial DNA divergence.

The difference in sequence between members of a family of transposons during the bursts is between approximately 1 percent and 14 percent, depending on the family. The burst of transposon SALT1, for example, initiates in salmon around 9 percent and ends around 6 percent (Fig. 3). On the other hand, the burst of DTSsa2, the most recently active transposon in salmon, lasts only from approximately 1.8 percent to 0.9 percent. Overall, these ranges of values of the overlapping bursts places these events after the assumed genome duplication in the salmonid line and well overlapping with later speciation events. These findings are in agreement with other studies that link transposon activity with large scale genomic reorganization. Different transposition mobilization patterns are observed in humans and chimpanzees after their separation from a common ancestor [44], and extensive transposition during genome stabilization following species hybridization has been documented [16,45].

## Conclusion

In conclusion, our data suggests that the timing of the bursts of transposon replication activity coincides with the time of radiation of the *Salmoninae *to *Salmo*, *Oncorhynchus*, and *Salvelinus *and subsequent speciation, as several waves of transposon activities sweep through the species. Our data supports the hypothesis that laterally injected massive transposon movement played a role in chromosomal reorganization at various times during speciation.

## Methods

The starting point for this *in silico *analysis were the sequences for the two known salmon DNA transposons SALT1 [Genbank:L22865] [19] and Tss [Genbank:L12207] [18], as well as an analysis of the sequence of the T-cell receptor alpha locus of *Salmo salar *by RepeatMasker [46]. These two transposons as well as the RepeatMasker data were used to find faint similarities which were used in turn to find a larger number of each family in approximately 3 Mbp of sequence. The Dotter program [47] was used extensively to find regions of similar sequence, which were extracted and stored in an SQL database. The length of the transposon sequences was determined by identifying the inverted terminal repeat sequences where possible. Sequence alignments were performed with ClustalW [48] and phylogenetic trees generated with MEGA3.1 [49] using the Unweighted Pair Group Method with Arithmetic Mean (UPGMA), pairwise deletion, and a p-distance model. The entire alignment of the sequences was used in the phylogenetic reconstruction. Our salmon EST database was searched for the presence of sequences that are similar to the DNA transposon sequences that we found in salmon.

The following DNA sequences and BAC clones were used in this analysis. The *Salmo salar *TCRα locus [30], the major histocompatibility loci MHC class 1a and 1b [29], the growth hormone and interleukin loci (manuscripts in preparation), and zoneadhesin-like genes [Genbank:AY785950] and the *Oncorhynchus mykiss *sequences for the metallothionein gene [GENBANK:DQ156151], MHC1a [Genbank:AB162342] and MHC1b loci [Genbank:AB162343], and the IgH.A locus [Genbank:AY872256]. Genbank sequence entries were used in this study from a variety of other organisms (table 2): *Oncorhynchus mykiss, Ictalurus punctatus, Esox lucius, Cyprinus carpio, Salvelinus namaycush, Salvelinus confluentus, Salvelinus fontinalis, Tanichthys albonus, Carassius auratus, Astatotilapia burtoni, Oryzias latipes, Petromyzon marinus, Danio rerio, Xenopus tropicalis, Xenopus laevis, Rana pipens*, and *Polypterus bichir*. Sequences from *Schistosoma japonicum *EST Genbank data were found for transposon families as follows: DTSsa1 [Genbank:AY915112, AY809993], DTSsa2 [Genbank:AY816058, AY834394], DTSsa3 [Genbank:AY124772], DTSsa4 [Genbank:AY812589, AY915240], DTSsa5 [Genbank:AY813498], DTSsa6 [Genbank:AY813020], DTSsa7 [Genbank:AY813225, AY915121], SSTN1 [Genbank:AY809988, AY815476, AY915835], Tss [Genbank:AY915400, AY915891], and SALT1 [Genbank:AY223470, AY915102].

Representative sequences from all new families have been deposited in GenBank under accession numbers EF685954 – EF685960, EF685962 – EF685963, and EF685966 – EF685967.

## Note added in Proof

After the manuscript was accepted, two additional DNA transposons were identified as follows: DTSsa15 [Genbank: EU147004] and DTSsa16 [Genbank: EU147005].

## Authors' contributions

JdB performed the analysis and drafted the manuscript.

RY performed DNA sequencing for the project.

WD contributed to the project planning and direction.

BK contributed to the planning, design, and direction of the project.

All authors read and approved the final manuscript

## Supplementary Material

Additional file 1 Sequences of the Tc1-like DNA transposons in multiple FASTA format. The data shows the DNA sequences of all the DNA transposons that were used in the analysis. Click here for file

Additional file 2 Sequences of the piggyBac-like DNA transposons in multiple FASTA format. The data shows the DNA sequences of all the piggyBac-like transposons that were used in the analysis Click here for file

Additional file 3 Alignment of the Tc1-like DNA transposon sequences in .aln format. The data shows the alignment of the Tc1-like sequences. Click here for file

Additional file 4 Alignment of piggyBac-like DNA transposon sequences in .aln format. The data shows the alignment of the piggyBac-like sequences. Click here for file
